# Peripheral Attentional Targets under Covert Attention Lead to Paradoxically Enhanced Alpha Desynchronization in Neurofibromatosis Type 1

**DOI:** 10.1371/journal.pone.0148600

**Published:** 2016-02-16

**Authors:** Gilberto Silva, Maria J. Ribeiro, Gabriel N. Costa, Inês Violante, Fabiana Ramos, Jorge Saraiva, Miguel Castelo-Branco

**Affiliations:** 1 ICNAS – Brain Imaging Network of Portugal, Coimbra, Portugal; 2 IBILI – Institute for Biomedical Imaging in Life Sciences, Faculty of Medicine, University of Coimbra, Coimbra, Portugal; 3 Medical Genetics Department, Pediatric Hospital of Coimbra, Coimbra, Portugal; 4 The Computational, Cognitive and Clinical Neuroimaging Laboratory, The Division of Brain Sciences, Imperial College London, London, United Kingdom; University of Zurich, SWITZERLAND

## Abstract

The limited capacity of the human brain to process the full extent of visual information reaching the visual cortex requires the recruitment of mechanisms of information selection through attention. Neurofibromatosis type-1 (NF1) is a neurodevelopmental disease often exhibiting attentional deficits and learning disabilities, and is considered to model similar impairments common in other neurodevelopmental disorders such as autism. In a previous study, we found that patients with NF1 are more prone to miss targets under overt attention conditions. This finding was interpreted as a result of increased occipito-parietal alpha oscillations. In the present study, we used electroencephalography (EEG) to study alpha power modulations and the performance of patients with NF1 in a covert attention task. Covert attention was required in order to perceive changes (target offset) of a peripherally presented stimulus. Interestingly, alpha oscillations were found to undergo greater desynchronization under this task in the NF1 group compared with control subjects. A similar pattern of desynchronization was found for beta frequencies while no changes in gamma oscillations could be identified. These results are consistent with the notion that different attentional states and task demands generate different patterns of abnormal modulation of alpha oscillatory processes in NF1. Under covert attention conditions and while target offset was reported with relatively high accuracy (over 90% correct responses), excessive desynchronization was found. These findings suggest an abnormal modulation of oscillatory activity and attentional processes in NF1. Given the known role of alpha in modulating attention, we suggest that alpha patterns can show both abnormal increases and decreases that are task and performance dependent, in a way that enhanced alpha desynchronization may reflect a compensatory mechanism to keep performance at normal levels. These results suggest that dysregulation of alpha oscillations may occur in NF1 both in terms of excessive or diminished activation patterns.

## Introduction

Neurofibromatosis type-1 (NF1) is the most frequent autosomal dominant neurogenetic disorder and a well-studied model of cognitive dysfunction with an average prevalence of 1 per 2500 to 3000[1]. It is caused by mutations in the neurofibromin gene and is characterized by multiple conditions, including benign tumors and neurological symptoms, associated with a myriad of behavioral manifestations[2,3]. The neurocognitive manifestations include learning disabilities, language and communication problems, attention and visuomotor integration deficits [3,4].

Given that attentional deficits are present in NF1 and that there is evidence of dysfunctional GABAergic signaling in patients affected by the disease [5], the NF1 phenotype is also a suitable model to study the role of inhibition in shaping alpha oscillations. Alpha oscillations (8-12Hz) are closely related to sensory input, but are also modulated by attention and relate to visual perception deficits [6–8].

The intriguing link between attention lapses that may occur even in healthy subjects and alpha oscillations has been demonstrated in target-detection tasks [9,10], revealing that increased power in the alpha band correlates with the probability of a subject missing a target [11]. In other words, higher vs. lower alpha activity are related with lower vs. higher visual performance levels, respectively. We have previously reported that alpha oscillations triggered by a low-level visual stimulus (centrally presented Gabor patches) were abnormally enhanced in NF1 [12]. These findings were interpreted in relation to the likelihood of missing upcoming targets (participants were instructed to report changes in the luminance of the fixation dot), which was found to be higher for individuals with NF1 than controls. The concurrency of increased alpha oscillations and reduced performance in an attentional task are in line with the studies mentioned above showing that, in essence, higher alpha during pre-stimulus periods is predictive of lower likelihood of detecting a target. On the other hand, during visual stimulation there is a normal pattern of decrease in parieto-occipital alpha activity i.e. alpha desynchronization or alpha suppression, interpreted as a sign of active neuronal processing. Moreover, alpha suppression can also result from directed attention, observed over the occipital region of the attended location [6,13]. The tight link between visual perception, attention and performance leads to the question whether alpha suppression may also be altered in NF1.

Here we tested the hypothesis of whether alpha oscillations elicited by a moving visual stimulus are also abnormally regulated when engaging covert attention in a population of patients with NF1. Previous studies have shown the importance of alpha waves in covert attention [10,14,15] and suggested a role for alpha oscillations in the top-down gating of anticipated stimuli [11,13,16–22]. In the present study, control children and children with NF1 performed a target detection task employing a visual stimulus consisting of non-centrally presented moving gratings. Participants were instructed to monitor the target, i.e. the peripherally presented gratings, throughout its duration and respond as fast as possible upon target offset. Non-central locations are directly related to exogenous attention mechanisms [23]. We studied oscillations triggered during the period subjects had to monitor the stimulus and produce a subsequent response. Our hypothesis was that NF1 subjects have difficulties in switching state mode: during rest it is harder for subjects to detect a target, if events such as its onset are unexpected and of short duration; and while observing a stimulus it is challenging to report target offset and switch afterwards to the rest mode. This would imply that subjects would actually show impaired attentional control both during rest (studied in Ribeiro et al. 2014 [12]) and during stimulation.

We found, surprisingly, an increased desynchronization of alpha power in patients with NF1, while recruiting covert attention upon target onset, combined with a relatively high accuracy. These findings suggest that a compensatory (or dysregulatory) mechanism controlling alpha activity may be at play and that disorders affecting attention, which is known to relate to alpha modulation, may be characterized by both increased and decreased activity, depending on context and performance.

## Methods

### Participants, recruitment and exclusion criteria

The study was composed of a total of forty-three children and adolescents (range: 9–19 years). Of these, twenty-seven were controls and sixteen were diagnosed with NF1 according to the National Institutes of Health Consensus Development Conference clinical criteria [24]. Participants with NF1 were recruited in collaboration with the Genetics Department of the Pediatric Hospital of Coimbra, Portugal.

Three control subjects were excluded due to the low number of artifact-free trials, twenty-four controls remained (16F/8M; mean age 13.47±2.74 years) as well as sixteen children and adolescents with NF1 (12F/4M; mean age 13.89±2.70 years). The age distribution (t = 0.33, DF = 38, n.s.) as well as gender distribution (*χ*^2^ = 0.193, DF = 2, n.s.) were not significantly different between groups. The Wechsler Intelligence Scale for Children (WISC-III, adapted to the Portuguese population)[25] was applied to a subgroup of participants and revealed a difference between groups in IQ (t = 3.24, df = 27, *P* = 0.003, IQ mean ± SD, control: 112 ± 3, N = 14, NF1: 96 ± 4, N = 15).

### Protocol approvals and patient consent

Informed consent was obtained from the participants’ legal representatives, after having explained the nature and purpose of the study. In addition, all participants gave written or oral consent. The study was conducted in accordance with the tenets of the Declaration of Helsinki and it was approved by the Ethics Committees of the Faculty of Medicine of the University of Coimbra and of the Children’s Hospital of Coimbra.

### Visual stimulation

**Fig 1:**
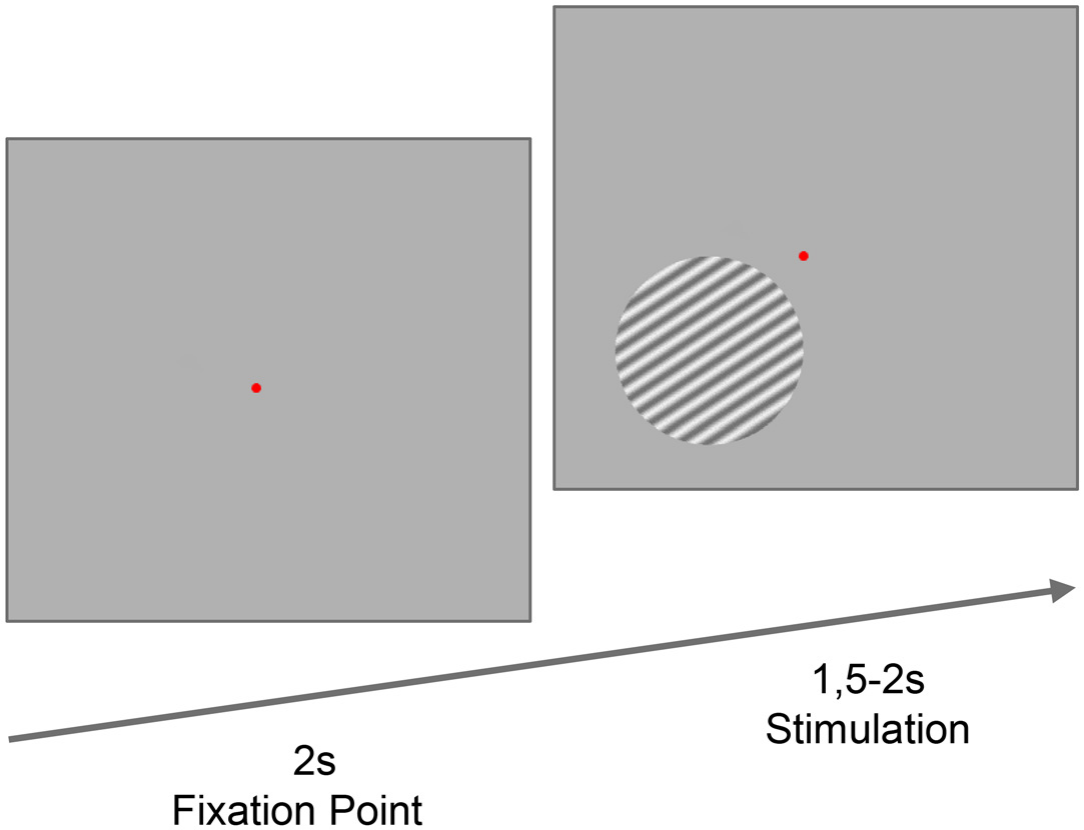
Visual Stimulus. Paracentral moving gratings recruiting exogenous attention. The moving grating is shown for a variable time (1500 ms-2000 ms) after a fixed interstimulus interval. Participants were instructed to maintain fixation in the central point during the whole task and to report the disappearance of the visual stimulus (target offset), as fast as possible. Two hundred trials were acquired, divided in two different runs, during which participants responded either with the right or the left hand.

The visual stimulus was adapted from Muthukumaraswami et al. *(2010)* [26] and consisted of a circular moving grating (80% contrast, spatial frequency 2 cycles/deg, 4 deg diameter, velocity 1 deg/second), presented against a grey background and with mean luminance equal to the background’s. Stimuli were presented in the lower left visual field, subtended 4 deg horizontally and vertically, with the center of the stimulus located 3.3 deg from the central fixation point. Stimulus duration was randomly defined between 1.5–2s. The stimulus presentation was followed by 2s when only the fixation point was shown (Fig 1). Participants were instructed to maintain fixation on the central point for the entire experiment and to press a response button as quickly as possible when the grating disappeared. The protocol consisted of two separate runs of 100 trials each, with participants responding with their right hand for the first run and with the left hand for the second, in a total of 200 trials for the final analysis.

### Behavioral analysis

We considered premature responses when participants responded until 150 ms after stimulus offset and misses if the response was absent. We did not perform statistical comparisons or correlation analyses with number of misses, as these represented very rare events (0.001% of total data). Participants were instructed to report the disappearance of the peripheral visual stimulus by pressing a button on a response pad.

### EEG acquisition and analysis

EEG was recorded using a 64-channel Neuroscan System (Neuroscan, v4.5), placed according to the International 10–20 system [27]. The reference and ground were positioned between CPz-Cz and Fz-FPz, respectively. The acquisition rate was 1000 Hz without any online filtering. Offline analysis was performed after down sampling the EEG data to 400 Hz in order to optimize data processing time. We applied a band pass hamming windowed FIR filter (1–100 Hz) and a notch filter (47.5–52.5 Hz). The datasets were divided into epochs locked to the beginning of the stimulus. To prevent contamination by blinks, the signal from ocular electrodes HEO and VEO was included in the automatic artifact rejection step. We rejected trials where the absolute amplitudes of EOG sensors exceeded 100 μV. For all the other channels, we scanned the entire dataset using a rejection threshold of 120 μV. We excluded from time-frequency analysis the trials where participants gave premature responses or when a response was absent. Participants with less than 30 artifact-free trials were excluded from further analysis (3 control participants were excluded). Bad channels were marked and interpolated using spherical spline interpolation. The available data after pre-processing represented above 70% of the total trials performed (control = 165±42 and NF1 = 134±46 of trials available for analysis). The HEO/VEO channels were excluded from further analysis and the recordings were re-referenced to the average of all channels.

Time-frequency analysis was performed using EEGLab functions (v12.0.2.5b) and custom Matlab scripts (version R2012b). Event related spectral perturbation (ERSP) was computed using Morlet wavelets with incremental cycles (3 cycles at 5Hz, up to 30 at 100Hz) resulting in 200 time points (from -500ms to 1500ms after stimulus onset) and 96 frequency points. The 500ms preceding the stimulus start were used as baseline (see S1 and S2 Figs for an analysis using an alternative baseline, showing virtually identical results). For the estimation of time-frequency spectra for each hemisphere, the posterior electrodes were divided into two different regional posterior clusters as units of measure (P1, P3, P5, P7, PO3, PO5, PO7, O1 and P2, P4, P6, P8, PO4, PO6, PO8, O2 respectively for the contralateral and ipsilateral sites), excluding those located at the central line between hemispheres (CPz, Pz, POz and Oz). The average posterior alpha and beta power was calculated including all the above mentioned posterior electrodes (i.e. of the right and left hemisphere) plus the posterior central line sensors (CPz, Pz, POz and Oz). Power values were obtained for each sensor and averaged over electrodes of interest for each subject for the cluster analysis.

In order to evaluate gamma oscillations (from 30Hz to 80Hz) variability, we compared post stimulus activity (from 50ms to 1500ms) from both groups using the maximum peak in the frequency spectrum power (i.e. the frequency in the gamma range where the participants had the maximum activation).

### Statistical analysis

Statistical analysis was computed on Matlab and IBM SPSS Statistics, v22 software. Normalcy of data was evaluated using the Shapiro-Wilk test. Data found not to have a normal distribution were analyzed with non-parametric tests (Mann-Whitney test). This was the case for premature responses (p<0.002 for both groups) while all the other parameters followed a normal distribution (p>0.05). Differences of alpha and beta power was evaluated using 2-way ANOVA with Bonferroni correction for multiple comparisons. We used Greenhouse-Geisser correction to avoid violations of sphericity in data analysis.

In order to evaluate lateralization differences, we compared the time-course activity of both hemispheres (paired statistics) and group (unpaired statistics) using cluster statistics based on Monte-Carlo estimates (1000 randomizations,), as described in Maris et al., 2007, [28]. Instead of a single sensor or frequency, we estimated differences using the regional cluster level as the unit of interest and for distinct frequency bands (average of separable frequency bands belonging either to the classical alpha or beta bands).

## Results

### Behavioral analysis

Participants were instructed to give a response as soon as the moving stimulus vanished (target offset). Response times and premature responses were analyzed for both groups. No significant group differences were found concerning reaction times (388.9±27.6 ms for controls, 427.2±26.4 ms for NF1, t = 0.953, df = 38, p>0.3). Premature responses were very infrequent (see methods) and both groups responded correctly in more than 95% of the trials. The number of premature responses was on average higher in the NF1 group, yet this difference was not significantly different (control≈3%, NF1≈8% of total responses, n.s.). Thus, the NF1 group revealed no overall hindrance in performing the task, with a total number of correct responses similar to that of control participants (control = 196.9±3.379 trials, NF1 = 192.2±9.123, p > 0.05).

### Analysis of pre-stimulus alpha power

**Fig 2:**
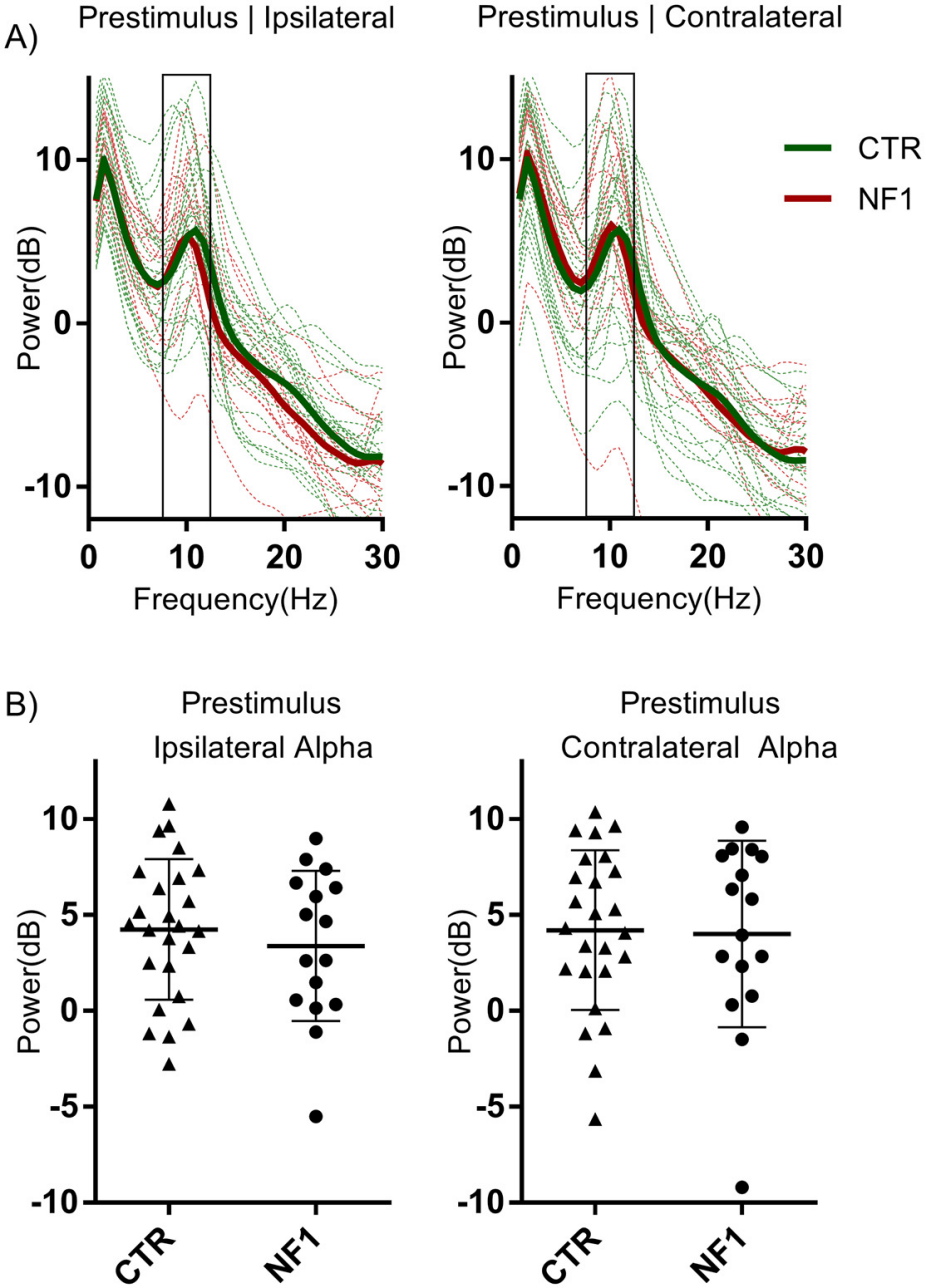
Average power spectrum for all participants. (A) Each line depicts average power for all channels of interest, for control (green) and NF1 (red) subjects. The solid lines show the average frequency power for each group in each condition. The rectangle represents the frequency band used to estimate the average alpha power (8-12Hz). (B) Average ipsilateral and contralateral alpha power for control (triangles) and NF1 (circles) subjects. Both groups show similar average alpha power (represented are mean ± SD).

The power of ongoing alpha oscillations (8-12Hz) was estimated from the pre-stimulus period (-500 ms to 0 ms), and focused on measurements of posterior regions, i.e. over visual processing brain areas. We analyzed the absolute power spectrum of the alpha band using the Fast Fourier Transform (FFT) to control for the possibility of an already altered basal state in oscillatory activity, with a particular focus on the alpha band. The power spectrum for participants of both groups revealed a typical alpha peak around 10 Hz (Fig 2A), for contralateral as well as ipsilateral occipito-parietal regions. No outliers (> mean ± 3*SD) were identified in any group. Regarding differences in pre-stimulus alpha power between control and patients with NF1, no significant differences were found between groups for contralateral (t = 0.035, DF = 38, control: 5.457 ± 0.8604 dB, NF1: 5.511 ± 1.407 dB) or ipsilateral (t = 0.45, DF = 38, control: 4.954 ± 0.7391 dB, NF1: 4.368 ± 1.131 dB) alpha power (Fig 2B).

### Alpha desynchronization

**Fig 3:**
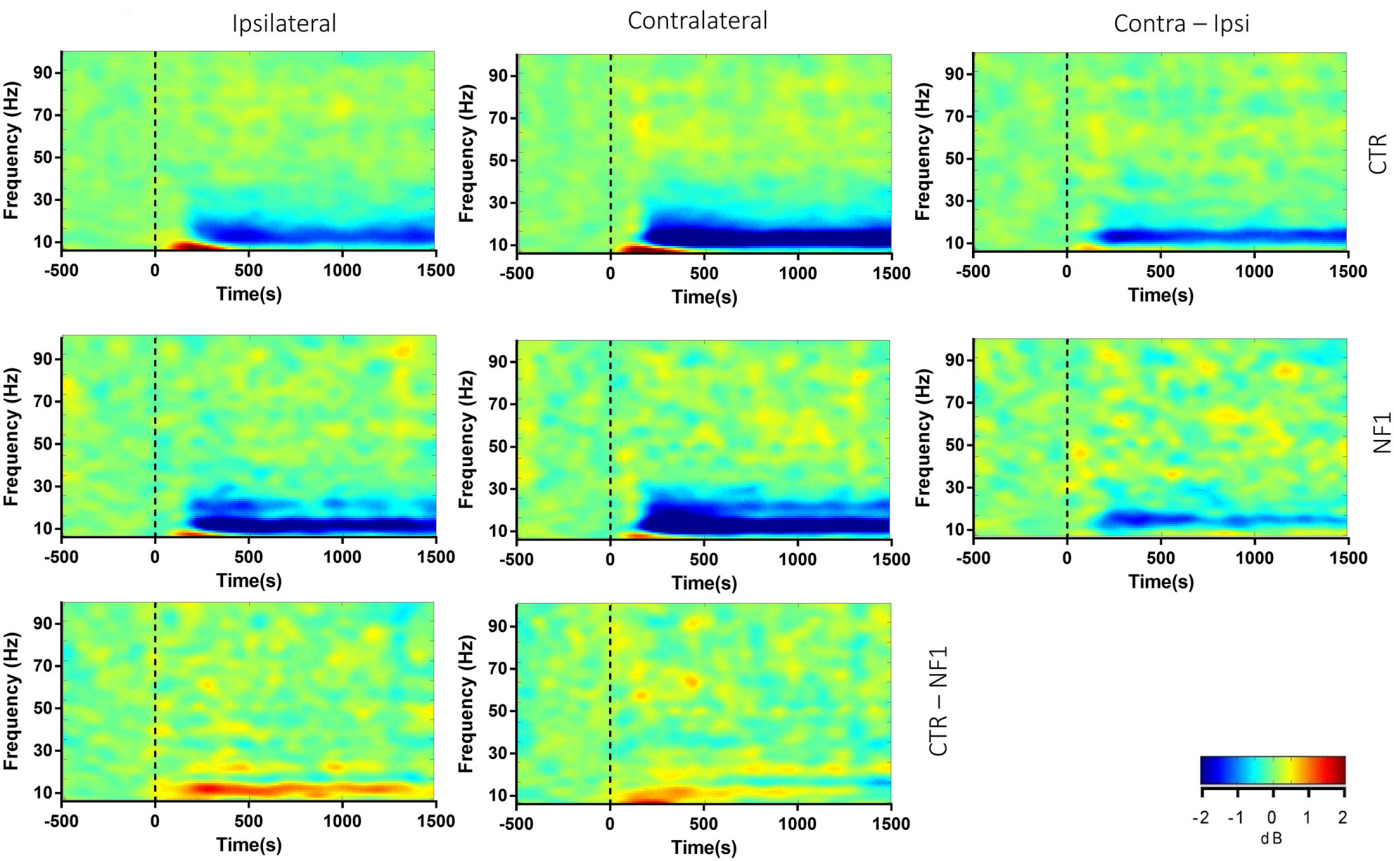
Time-frequency plots for the two studied groups. Posterior sites revealed high alpha deactivation, starting around 200 ms. The contralateral hemisphere exhibited higher deactivation, reflecting a retinotopic/hemisphere specific response to the stimulus. Deactivation in the ipsilateral hemisphere of NF1 participants is more evident than controls (see text for details). A beta specific band has a sustained deactivation starting at 200ms, stronger in the NF1 group. Changes in gamma frequencies in response to the stimulus are less evident. Differences between hemispheres within a group (third column, contra—ipsi) and between groups for each hemisphere (third row, CTR—NF1) are also shown and highlight the ipsilateral alpha deactivation observed in NF1.

The modulation of alpha power resulting from the presentation of the peripheral visual stimulus was estimated, and differences between groups in alpha power were evaluated across posterior cluster. Fig 3 shows the average event-related spectral perturbation (ERSP) for each group as time-frequency plots. In both control and NF1, decreases in power in the alpha band were evident after stimulus onset, as expected. This alpha suppression is more pronounced for the contralateral hemisphere, in both groups, but can also be found in the ipsilateral hemisphere.

**Fig 4:**
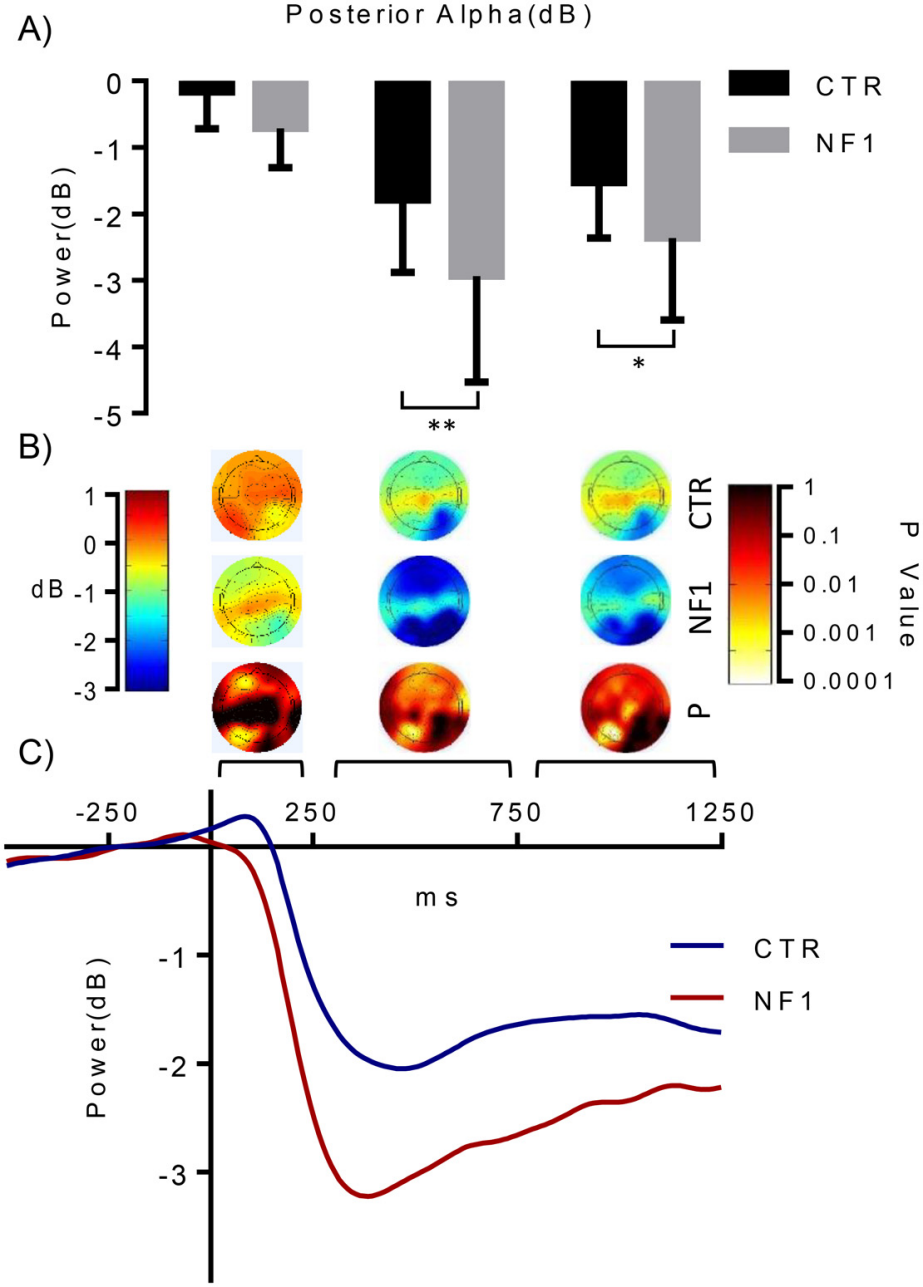
Time course and topography of event related alpha power. (A) Significant differences in alpha power were found at different time periods: 250-750ms and 750-1250ms in the posterior sites between control and NF1 subjects. (B) Head topography of alpha over early (0ms-250ms), middle (250-750ms) and late (750ms-1250ms) periods (C) The time course indicates deactivation starting around 150ms, reaching a minimum power peak around 300ms. The higher level of alpha deactivation found in NF1 participants, highlights the abnormal alpha modulation in these patients.

The analysis of the difference in alpha power between the two hemispheres (Fig 3, contra—ipsi) showed greater hemispheric differences for the control than NF1 group Event-related alpha desynchronization, here referred to as deactivation of neuronal oscillatory ensembles in the local sensor area (see Pfurtscheller et al., 1999 [29]), was also greater in the ipsilateral hemispheres of subjects with NF1 than controls (Fig 3). Significant differences in alpha power were found between the two hemispheres in the control group but not for the NF1 group (cluster permutation statistics, contralateral vs ipsilateral hemisphere, p<0.01; S3 Fig). Accordingly, differences in alpha activity between groups were found in the ipsilateral hemisphere (p<0.01), suggesting a partial reduction in the asymmetrical modulation of alpha activity in the NF1 group (see S3 Fig). For the two groups, there is a visible decrease in alpha power that is persistent throughout the stimulus presentation and more pronounced at posterior regions (Fig 4). The analysis of visually triggered alpha activity was carried out for the selected ipsilateral and contralateral electrodes described above, as well as the central line electrodes (CPz, Pz, POz and Oz). When comparing the posterior cluster between groups, we observed significant activity differences at the alpha band level, with NF1 patients showing a higher alpha desynchronization than controls (Fig 4A). Maximum differences between groups are at the interval 250-750ms (F _(1, 38)_ = 8.771, p<0.01, Bonferroni-corrected for multiple comparisons) and 750-1250ms (p<0.05, Bonferroni-corrected for multiple comparisons). Overall, a greater reduction in alpha power was observed for posterior electrodes of NF1 subjects than controls (see Fig 4B). Event related alpha response plots confirm this pattern: a sustained deactivation starting after 150ms and peaking around 300ms (Fig 4C). These results were not influenced by the choice of baseline time window (see S1 and S2 Figs).

### Beta band activity

**Fig 5:**
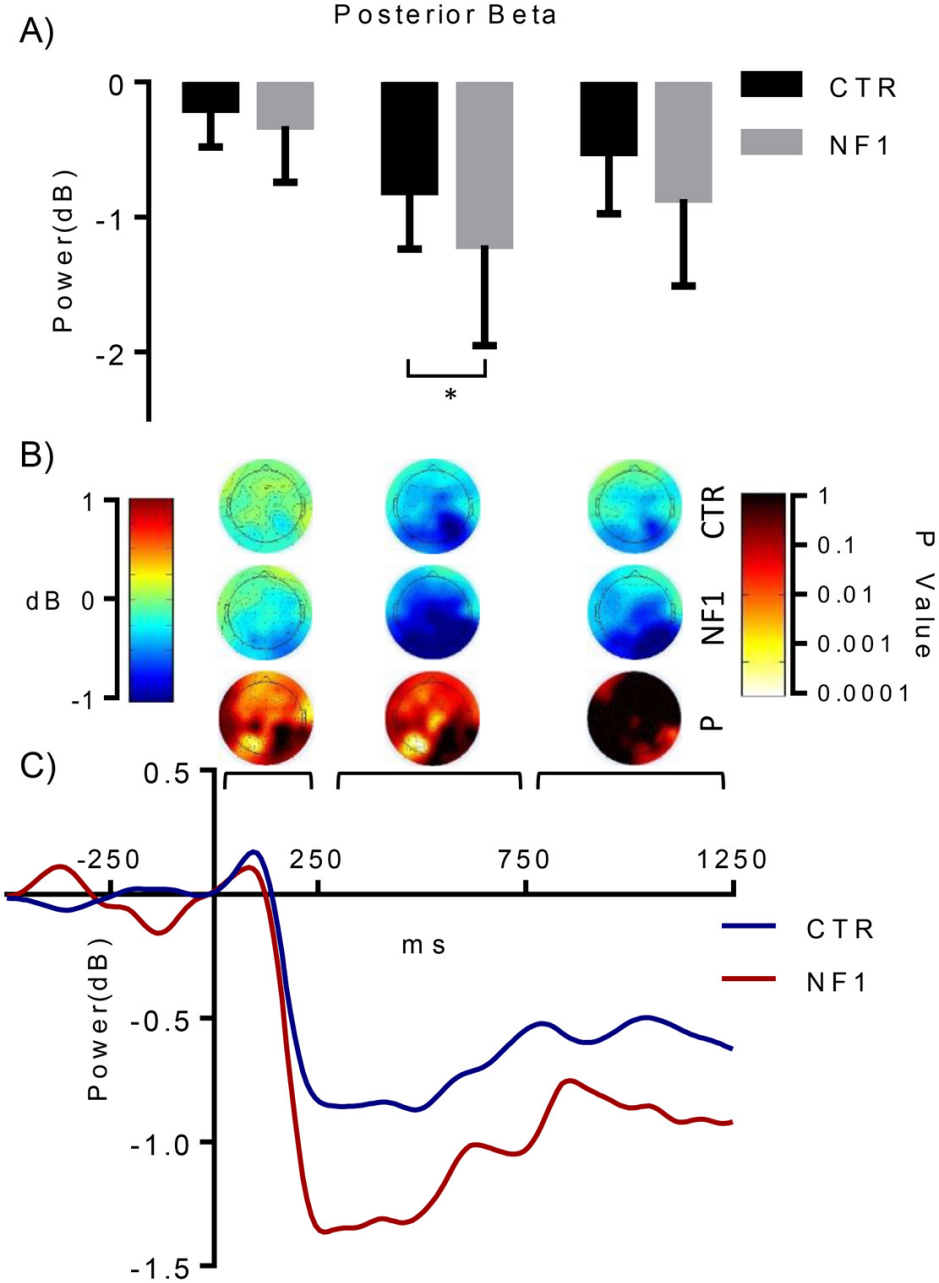
Time course and topography of beta power. (A) NF1 children revealed abnormal beta deactivation in posterior sites. Specific time periods (250-750ms) revealed significant differences between groups. (B) Topographies showed beta deactivation to be more evident and broadly distributed over posterior electrodes on NF1 participants, while control subjects showed a slightly higher deactivation in the contralateral site. (C) The time course Indicated deactivation starting around 150ms, reaching a minimum power peak around 300ms. Beta deactivation is stronger in NF1 participants, suggesting a greater event-related reduction of beta power in this group.

Posterior activity at beta frequencies showed a similar pattern as that of alpha. The time-frequency plots revealed a specific separable beta sub-band (17–23 Hz) showing a stable and sustained deactivation particularly in NF1 (Fig 3). This beta deactivation was more pronounced at the contralateral hemisphere (Fig 5), but was not so evident in controls. Differences between control and NF1 were only found for posterior beta power 250 to 750ms after stimulus onset (F _(1, 38)_ = 4.483, p < 0.05, Bonferroni-corrected). This suggests that beta oscillations related to sensory and attentional processing might also be altered in NF1.

### Gamma band activity

**Fig 6:**
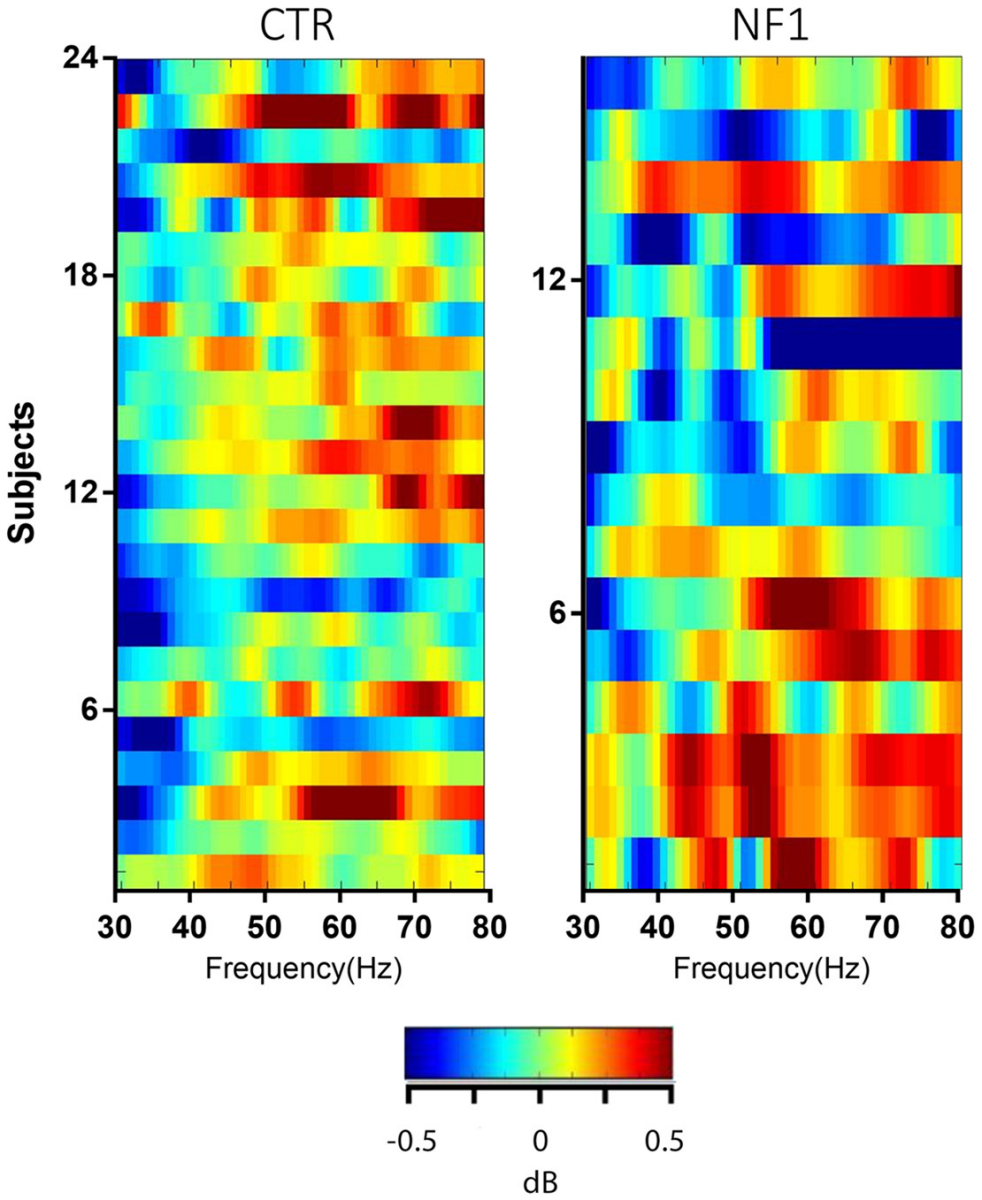
Single subject analysis of gamma power. Average power values for gamma frequencies (averaged over 50ms-1500ms) for each participant. Despite a higher variability of gamma activity observed for the NF1 group, no particular pattern of gamma activity is discernible in the two groups.

The activity at gamma frequencies was overall less conspicuous from the group average time-frequency plot of both control and NF1 (Fig 3). We hypothesized that this less obvious pattern could be the result of a considerable “averaging out” the activity due to inter-subject variability in the frequencies of peak gamma activity. In fact, previous reports show persistent activity at subject-specific gamma sub-bands in similar visual paradigms [30,31]. In the visual cortex, visual stimulation has been shown to elicit a reduction in alpha oscillations amplitude with concomitant increases of activity in the gamma band [32]. For this reason, an effort was made to identify predominant gamma sub-bands for each subject for gamma frequencies ranging from 30 Hz to 80 Hz but no consistent activity pattern was found (Fig 6). This analysis was carried out by averaging the post-stimulus/event-related gamma power over the time dimension, for each participant. The distribution of gamma activity was found to be widely scattered among subjects of both groups, with no consistent peaks of activity across all subjects. In any case, it was clear that subjects with NF1 show much more variability in gamma peaks than controls. We found no significant differences in the mean frequency of the gamma peaks between groups (55.63±12.43 Hz for control group and 56.13±10.79 Hz for NF1 group, DF = 38, ns) and no within subject consistent peak of gamma modulation.

## Discussion

The present work addressed how peripheral stimuli under covert attention conditions modulate brain activity patterns in NF1, a model of impaired inhibitory neurotransmission. We had previously demonstrated enhanced modulation of alpha oscillations triggered by centrally presented Gabor patches [12], under conditions where participants were asked to respond as soon as they detect an upcoming target onset.

Here, a moving grating was used as a cue to which participants had to allocate their attention to and provide a response upon its disappearance (hence, responding to the target offset). By presenting the stimulus outside the central visual field, covert attention mechanisms that require more significantly top-down control could be probed. Moreover, by asking participants to respond only after the target stimulus had ceased, we investigated the functional significance of altered oscillatory patterns triggered by the prior onset of that target stimulus in relation to attentional recruiting. In this way, the present task (detection of target offset) required disengagement from the state triggered by monitoring of a prior stimulus so that a fast and reliable response could be executed. While presenting the visual stimulus to only the left side might be a limitation of the present study, the use of a more elaborate visual stimuli, such as an unpredictable grating position, would increase the number of tested conditions, reducing the number of trials available for each condition. This is an issue that should nonetheless be addressed in future studies.

Alpha rhythms are related to activity in visual thalamocortical circuits [33,34], as well as perception and attention [6–8,14,16,19,35–38]. Broad attentional deficits have been so far related to an overall increase of alpha oscillations, which is associated to unfocused, underperforming states. Here we found an additional abnormal mechanism, whereby alpha desynchonization triggered by a visual stimulus is increased in patients. A dichotomic dysregulatory pattern is therefore possible. The enhanced alpha desynchronization observed in here while monitoring a target and detecting its offset might be explained in the context of abnormal attentional regulation. Since high alpha power is generally related to unfocused states, we speculate that low alpha during monitoring may relate to enhanced compensatory focusing in patients to allow for normal performance levels. In any case, it is clear that in NF1 both excessive and diminished alpha may occur, suggesting abnormal regulation of these oscillatory patterns.

Previous studies have focused mainly on alpha oscillations during the resting state and the anticipatory behavioral changes associated to them [6,11,13,16,20,21]. Alpha power levels prior to stimulus presentation are negatively correlated with the probability of detecting an upcoming stimulus [20], and can in fact be used to predict lapses of attention [11]. During visual stimulation, a reduction in alpha power is expected, in agreement with the sensorial-gating hypothesis of alpha [6]. Indeed, we found alpha suppression in both control and children with NF1 while the stimulus was present and prior to target offset. Alpha deactivation was found to be greater for the NF1 group after the stimulus onset when compared to the alpha activity observed in control subjects. The stronger alpha deactivation in NF1 participants may reflect abnormal regulation of exogenous attention in response to the target stimulus. Given that performance was preserved (except for the number of premature responses), such abnormal regulation is not necessarily maladaptive and might actually reflect an overcompensating process. Such mechanism would explain why we found a stronger decrease in alpha power in participants with NF1 under relatively preserved performance, in contrast with previous findings of increased alpha oscillations in underperforming subjects with NF1 [12].

We found also evidence for group differences in the lateralization of alpha suppression. We expected that suppression would retinotopically match the posterior regions in the right hemisphere, but in subjects with NF1 alpha power decrease in the ipsilateral site of stimulation was similar to that observed over contralateral regions (see Fig 3). Accordingly, compared with controls, participants with NF1 revealed significantly higher desynchronized alpha in the ipsilateral hemisphere and only the control group did exhibit significant inter-hemispheric alpha power differences in the posterior area.

Differences in activity at a specific beta band range (17–23 Hz) were also evident between groups. The role played by beta oscillations in visual processing is not well understood and the observed changes are consistent with studies supporting an important role for beta band modulation in sensorimotor control as well as attention [39,40] (see Fig 5). We believe that this difference is not simply the result of frequency bleeding since, despite the similar time-course observed for beta and alpha power, distinct topographies were observed for the two frequency bands (Figs 4 and 5) and since there is a visible separation of around 5 Hz between the modulated alpha band and the beta band in question (visible mainly in NF1 time-frequency spectra, Fig 3).

It is known that posterior alpha activity is closely related to visual processing [7,10,41,42] and to visual spatial attention [15]. Moreover, alpha suppression is correlated to increased gamma activity [32], which suggests a gating function of alpha supported by the inverse correlation of and dissociation between alpha and gamma power [32,43]. Gamma activity is often associated with sensory processing, including vision [44], but also in perception and higher cognitive functions such as attention and binding [17,45,46]. Findings of dysfunctional gamma in neurodevelopmental disorders such as autism [47,48], suggest that they may also have a role in these impaired cognitive functions. GABAergic signaling, which is affected in NF1 [5], has been shown to correlate with gamma activity [30]. Moreover, gamma and alpha oscillations are closely related with attention and visual perception deficits [7,8,11]. For these reasons, we also explored stimulus dependent changes in gamma activity in NF1. While changes in gamma activity have been reported in autistic spectrum disorders [49], for which NF1 is a suitable model, we did not observe similar changes in the present study. While gamma activity at specific frequencies (mostly reported between 55–80 Hz) can be discerned in a few subjects, no consistent gamma pattern can be found for either group, precluding an analysis comparing gamma activity (see Fig 3 for the group average ERSP’s).

We conclude that alpha band dysregulation in NF1 can manifest itself both in terms of excessive as well as reduced power (as found in the present paper), in a task and attentional state dependent manner. Future studies should address whether such abnormal pattern is also present in other developmental disorders such as autism, and its relation with unfocused vs. hyper focused attentional processes. These should also integrate stimuli patterns designed to test additional types of attentional processes, rather than only exogenous attention. The fact that enhanced desynchronization is coupled with relatively preserved performance, suggesting the presence of compensatory mechanisms, is consistent with the proposal that training alpha modulation by means of Brain Computer Interface and Neurofeedback approaches are worthy endeavors [50,51].

## Supporting Information

S1 Fig Time-frequency analysis using different baseline periods. Time-frequency spectra of posterior cluster for both groups: control and NF1 estimated relative to two baseline time windows: from -500ms to 0ms (original); and from -500ms to -150ms (adjusted), to exclude a contribution of post-stimulus power changes contaminating the original baseline (ending at 0 s). As can be seen, the results obtained are the same regardless of the selected baseline period.
(TIF)

S2 Fig Posterior alpha power time-course using distinct baseline periods. Comparison of time-course of alpha power for two different baselines. The full lines are superimposed with dashed lines, comparing the two baseline designs (from -500ms to 0ms and -500ms to -150ms, respectively) and show that the two distinct baseline computations lead to the same results.
(TIF)

S3 Fig Inter-hemispheric asymmetry patterns. Time-frequency spectra for control and NF1 participants, divided by hemispheres. Time-courses denote the average power of the canonical alpha band (8-12Hz) along time points. Differences between hemispheres and groups in alpha power time-course were evaluated using cluster permutations, p<0.01 (significant differences are depicted by the grey bar on the top of time-course graphs). CTR and NF1 groups are represented in green and red, respectively, while full and dotted lines represent contralateral and ipsilateral sites. The black dashed lines represent the difference between NF1 and CTR (bottom row) or between hemispheres (right column) within groups. The Ipsilateral hemisphere of NF1 shows more prominent desynchronization than the control group. Furthermore, there is a significant difference between hemispheres in the control group.
(TIF)
