# Supplementary figures and images for: Peripheral Attentional Targets under Covert Attention Lead to Paradoxically Enhanced Alpha Desynchronization in Neurofibromatosis Type 1
Source: PLoS One. 2016 Feb 16;11(2):e0148600. doi: 10.1371/journal.pone.0148600 (PMC4755663; doi:10.1371/journal.pone.0148600)

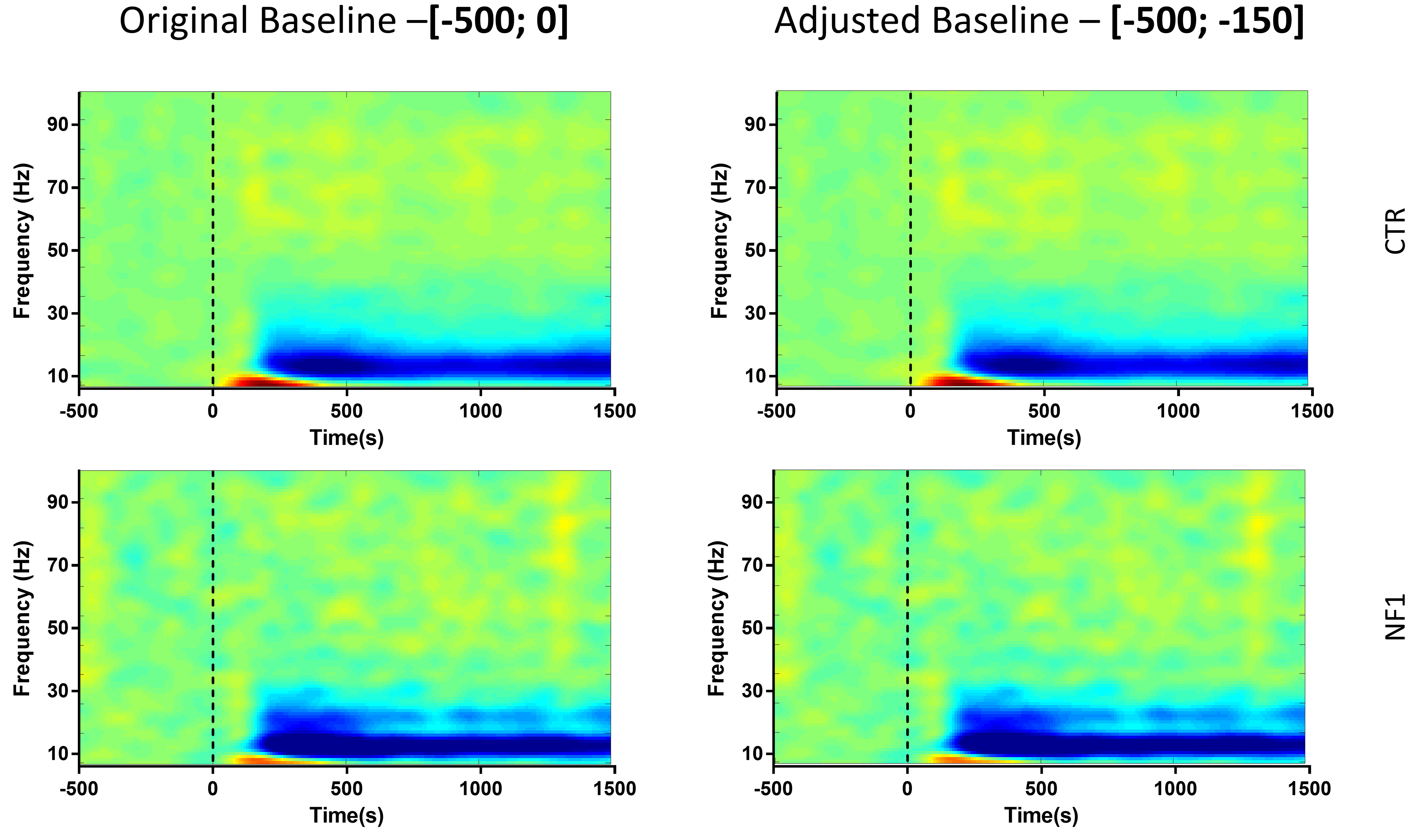

Supplement: S1 Fig — Time-frequency spectra of posterior cluster for both groups: control and NF1 estimated relative to two baseline time windows: from -500ms to 0ms (original); and from -500ms to -150ms (adjusted), to exclude a contribution of post-stimulus power changes contaminating the original baseline (ending at 0 s). As can be seen, the results obtained are the same regardless of the selected baseline period. (TIF) [file pone.0148600.s001.tif]

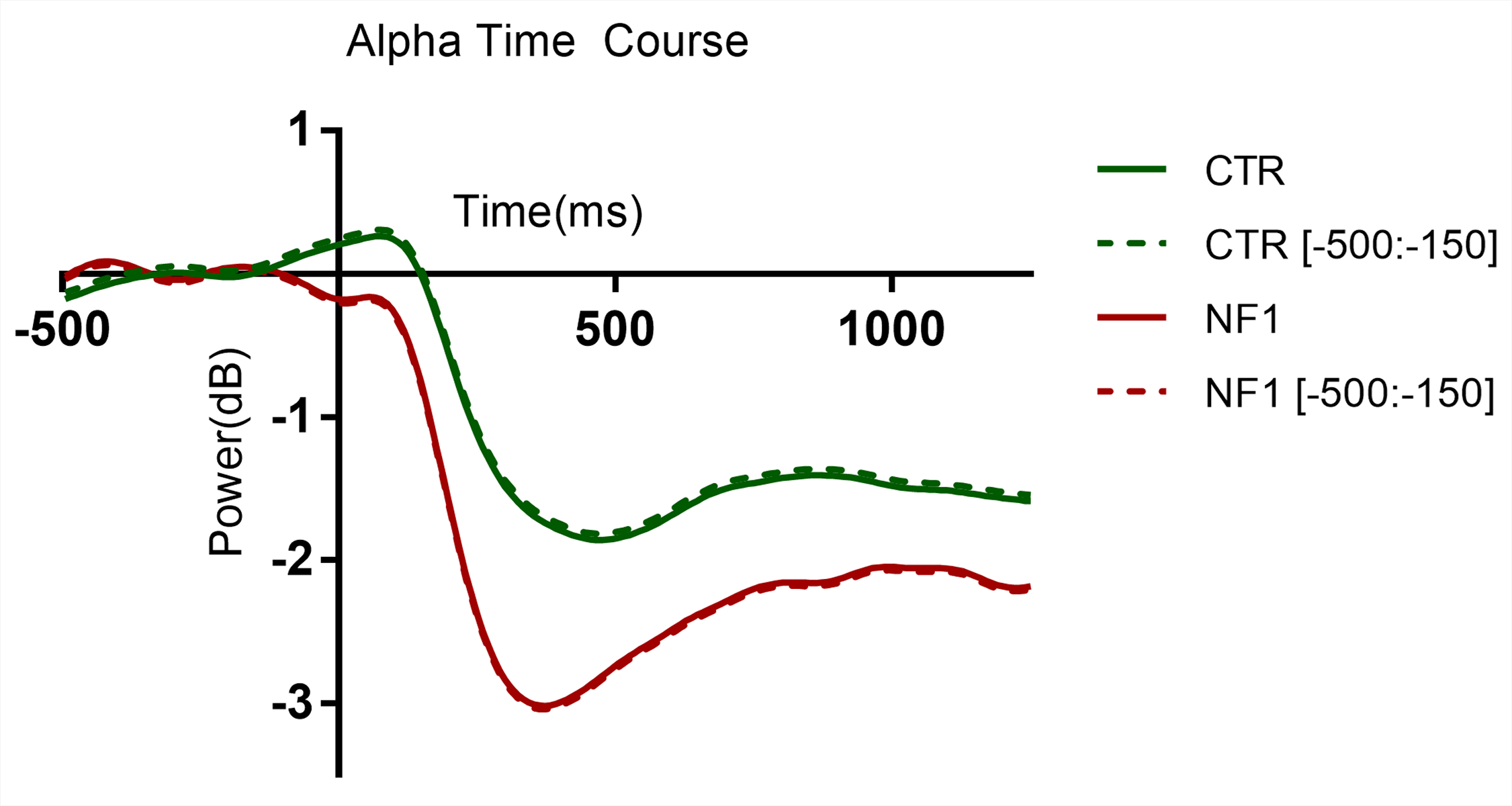

Supplement: S2 Fig — Comparison of time-course of alpha power for two different baselines. The full lines are superimposed with dashed lines, comparing the two baseline designs (from -500ms to 0ms and -500ms to -150ms, respectively) and show that the two distinct baseline computations lead to the same results. (TIF) [file pone.0148600.s002.tif]

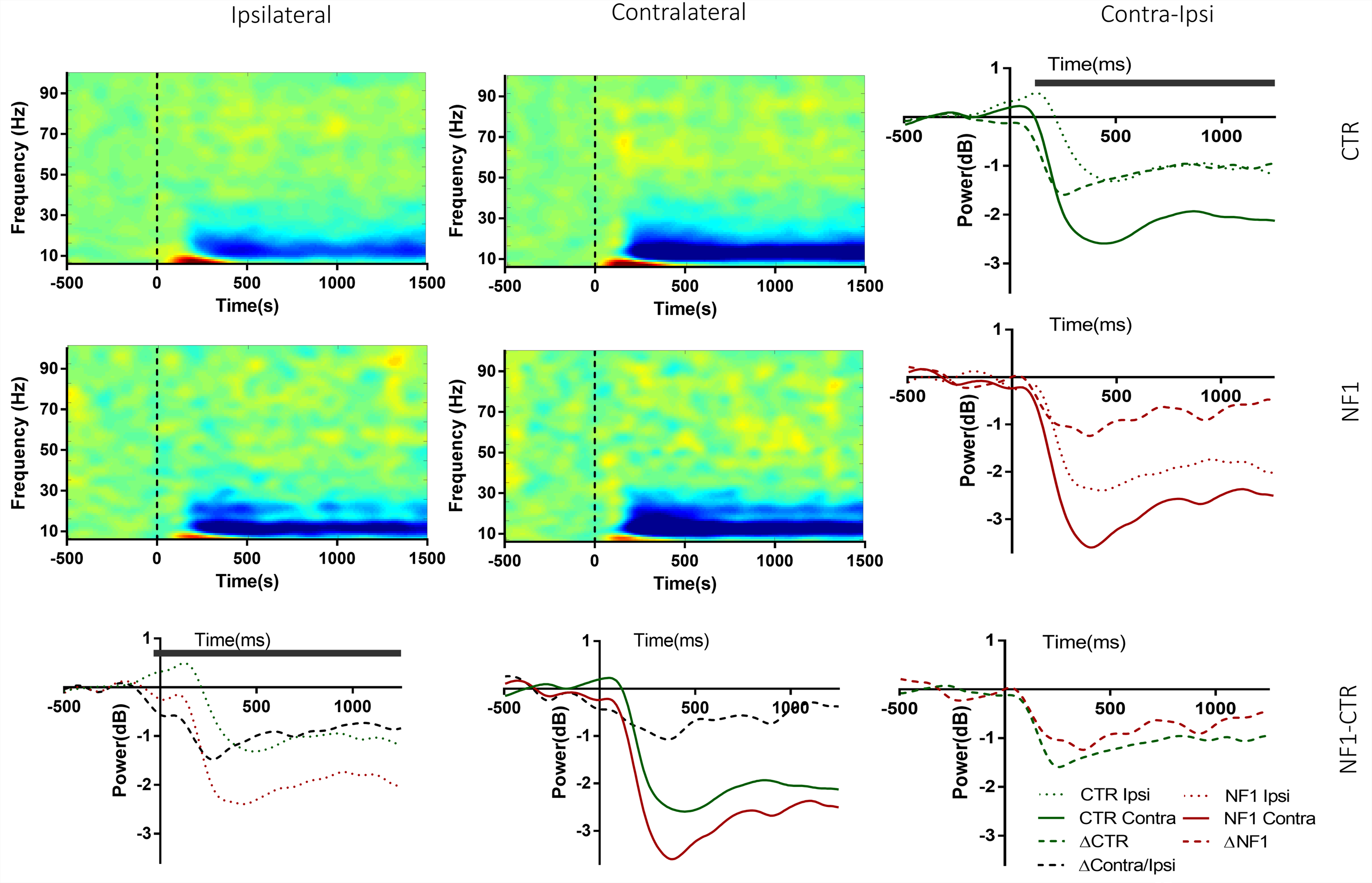

Supplement: S3 Fig — Time-frequency spectra for control and NF1 participants, divided by hemispheres. Time-courses denote the average power of the canonical alpha band (8-12Hz) along time points. Differences between hemispheres and groups in alpha power time-course were evaluated using cluster permutations, p<0.01 (significant differences are depicted by the grey bar on the top of time-course graphs). CTR and NF1 groups are represented in green and red, respectively, while full and dotted lines represent contralateral and ipsilateral sites. The black dashed lines represent the difference between NF1 and CTR (bottom row) or between hemispheres (right column) within groups. The Ipsilateral hemisphere of NF1 shows more prominent desynchronization than the control group. Furthermore, there is a significant difference between hemispheres in the control group. (TIF) [file pone.0148600.s003.tif]
